# Structural Basis of Nanobodies Targeting the Prototype Norovirus

**DOI:** 10.1128/JVI.02005-18

**Published:** 2019-03-05

**Authors:** Kerstin Ruoff, Turgay Kilic, Jessica Devant, Anna Koromyslova, Alessa Ringel, Alexander Hempelmann, Celina Geiss, Juliane Graf, Michelle Haas, Imme Roggenbach, Grant Hansman

**Affiliations:** a Schaller Research Group at the University of Heidelberg and the DKFZ, Heidelberg, Germany; b Department of Infectious Diseases, Virology, University of Heidelberg, Heidelberg, Germany; c Max Planck Institute for Molecular Genetics, Berlin, Germany; University of Pittsburgh School of Medicine

**Keywords:** X-ray crystallography, norovirus

## Abstract

Human norovirus infections are a major disease burden. In this study, we analyzed three new norovirus-specific Nanobodies that interacted with the prototype human norovirus (i.e., genogroup I genotype 1 [GI.1]). We showed that the Nanobodies bound on the side (Nano-7 and Nano-62) and top (Nano-94) of the capsid-protruding (P) domain using X-ray crystallography. Nano-7 and Nano-62 bound at a similar region on the P domain, but the orientations of these two Nanobodies clashed with the shell (S) domain and neighboring P domains on intact particles. This finding suggested that the P domains on the particles should shift in order for Nano-7 and Nano-62 to bind to intact particles. Interestingly, both Nano-7 and Nano-94 were capable of blocking norovirus virus-like particles (VLPs) from binding to histo-blood group antigens (HBGAs), which are important cofactors for norovirus infection. Previously, we showed that the GI.1 HBGA pocket could be blocked with the soluble human milk oligosaccharide 2-fucosyllactose (2′FL). In the current study, we showed that a combined treatment of Nano-7 or Nano-94 with 2′FL enhanced the blocking potential with an additive (Nano-7) or synergistic (Nano-94) effect. We also found that GII Nanobodies with 2′FL also enhanced inhibition. The Nanobody inhibition likely occurred by different mechanisms, including particle aggregation or particle disassembly, whereas 2′FL blocked the HBGA binding site. Overall, these new data showed that the positive effect of the addition of 2′FL was not limited to a single mode of action of Nanobodies or to a single norovirus genogroup.

**IMPORTANCE** The discovery of vulnerable regions on norovirus particles is instrumental in the development of effective inhibitors, particularly for GI noroviruses that are genetically diverse. Analysis of these GI.1-specific Nanobodies has shown that similar to GII norovirus particles, the GI particles have vulnerable regions. The only known cofactor region, the HBGA binding pocket, represents the main target for inhibition. With a combination treatment, i.e., the addition of Nano-7 or Nano-94 with 2′FL, the effect of inhibition was increased. Therefore, combination drug treatments might offer a better approach to combat norovirus infections, especially since the GI genotypes are highly diverse and are continually changing the capsid landscape, and few conserved epitopes have so far been identified.

## Abstract (precis)

The discovery of vulnerable regions on norovirus particles is instrumental in the development of effective inhibitors, particularly for GI noroviruses that are genetically diverse. Analysis of these GI.1-specific Nanobodies has shown that similar to GII norovirus particles, the GI particles have vulnerable regions. The only known cofactor region, the HBGA binding pocket, represents the main target for inhibition. With a combination treatment, i.e., the addition of Nano-7 or Nano-94 with 2′FL, the effect of inhibition was increased. Therefore, combination drug treatments might offer a better approach to combat norovirus infections, especially since the GI genotypes are highly diverse and are continually changing the capsid landscape, and few conserved epitopes have so far been identified.

## INTRODUCTION

Human norovirus is recognized as the most important cause of outbreaks of acute gastroenteritis (1). Currently, there are no available vaccines or antivirals for noroviruses, despite their discovery decades ago (2). Norovirus is a nonenveloped single-stranded RNA virus within the *Caliciviridae* family. The genome contains three open reading frames (ORFs), where ORF1 encodes nonstructural proteins, ORF2 encodes the major capsid protein (VP1), and ORF3 encodes the minor capsid protein (VP2). Based on the capsid gene sequences, noroviruses can be divided into at least seven genogroups (GI to GVII), with GI, GII, and GIV causing infections in humans. Each human genogroup is further subdivided into numerous genotypes (3).

The norovirus virion comprises 90 VP1 dimers that form an icosahedral particle (T=3) 35 to 45 nm in diameter (2, 4). The capsid protein can be expressed in insect cells and self-assembles into virus-like particles (VLPs) assumed to be morphologically similar to native virions. The X-ray crystal structure of prototype genogroup I genotype 1 (GI.1) norovirus VLPs showed that the capsid is divided into two domains, the shell (S) and protruding (P) domains, which are connected via a flexible hinge (4). The S domain forms the scaffold surrounding the RNA, while the surface-exposed P domains, which are further subdivided in P1 and P2 subdomains, contain the main determinants of antigenicity and host binding epitopes. Norovirus interaction with cofactor histo-blood group antigens (HBGAs) is important for infection (e.g., GI.1, GII.4, GII.10, and GII.17), although certain genotypes poorly bind HBGAs (e.g., GII.1) (5–8). HBGAs are found as soluble antigens in saliva and are expressed on epithelial cells. Studies have indicated that norovirus may interact with HBGAs prior to cell attachment (9) and/or bind particles on cell surfaces (10).

A recent study showed that human monoclonal antibodies (MAbs) targeting the GI.1 HBGA pocket inhibited norovirus VLPs from binding to HBGAs by steric interference with the HBGA pocket (11). In our previous studies, we identified norovirus-specific single-chain variable domains (Nanobodies) that block GII norovirus VLP binding to HBGAs (13, 20). We also showed that human milk oligosaccharides (HMOs), e.g., 2-fucosyllactose (2′FL), block the GI.1, GII.4, GII.10, and GII.17 HBGA binding pockets (12, 14–16). HMOs are the third most abundant compound of human milk and were shown to protect against various pathogens (17, 18). HMOs structurally resemble HBGAs, both being complex glycans that consist of differently linked monosaccharides. HMOs are thought to act as a receptor decoy by mimicking HBGAs and thus blocking virus attachment (19).

As with many antivirals, insufficient cross-reactivity among the diverse norovirus genotypes appears to be a limiting factor for broad-range therapy (11, 20). For other viruses, enhanced effectivity has been achieved by joint administration of several compounds. Indeed, current state-of-the-art therapies take advantage of drug combination approaches. Through additive or synergistic effects, the administration of a combination of drugs can enhance the effectiveness without increasing the overall dose. The drugs can interact to magnify their effects, or, by independently acting on different target sites, ultimately the compounds can complement each other to maximize the response rate to generate a highly efficient combination therapy. In the current standard treatment guidelines for HIV infections, three synergistically acting drugs are used. These compounds are from different drug classes and target distinct stages of the virus life cycle to suppress virus proliferation (21–23). Investigation of synergistic or additive drug effects is common in antiviral research for a variety of viruses, such as hepatitis B virus (HBV) and dengue virus (19, 24, 25). Additionally, several studies have shown synergistic effects for viral inhibitors targeting the stage of viral attachment. Combinations of different neutralizing MAbs exhibited positive effects against a number of different viruses, such as HIV, hepatitis C virus, and severe acute respiratory syndrome (SARS) coronavirus (26–29). Furthermore, synergistic inhibition of HIV has been observed for combinations of soluble forms of the receptor CD4 combined with different neutralizing MAbs (30). Combined antiviral activity of drugs was also demonstrated in studies using rotavirus, another common cause of gastroenteritis. In these studies, combinations of plant extracts or plant-derived compounds were shown to synergistically increase the inhibition of rotavirus replication *in vitro* (31, 32).

In the current study, we determined the X-ray crystal structures of three novel GI.1-specific Nanobodies (Nano-7, Nano-62, and Nano-94) in complex with the GI.1 P domain. We showed that Nano-7 and Nano-62 bound on the side of the P domain, whereas Nano-94 bound on the top. Our data suggest that Nano-7 and Nano-62 might clash with the S domain and neighboring P domains, which would mean that the P domains on particles would need to shift to accommodate Nanobody binding. Interestingly, Nano-7 and Nano-94 also blocked VLPs from binding to HBGAs. More importantly, we discovered that a combination of either Nano-7 or Nano-94 with 2′FL improved HBGA binding inhibition.

## RESULTS

### Nanobody binding to P domain and VLPs.

**FIG 1:**
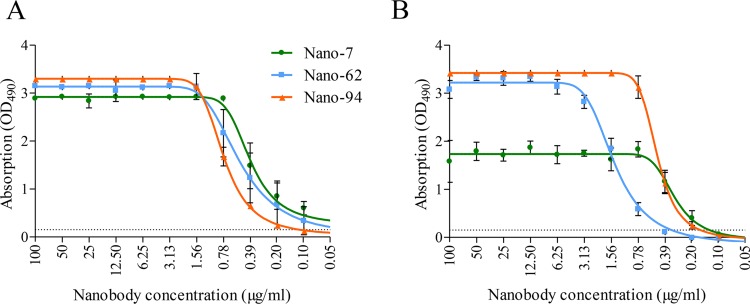
Nanobody binding to GI.1 capsid. The P domain or VLPs were coated on plates and detected with serially diluted Nanobodies. Experiments were performed in triplicate (error bars shown), and the cutoff was set at OD_490_ of 0.15 (dashed line). (A) All three Nanobodies were capable of detecting 10 μg/ml of GI.1 P domain in a dose-dependent manner, where Nano-7, Nano-62, and Nano-94 detection levels were 0.2 μg/ml, 0.1 μg/ml, and 0.1 μg/ml, respectively. (B) All three Nanobodies bound to 5 μg/ml of GI.1 VLPs, although for Nano-7, the maximum OD_490_ signal was approximately 2-fold lower than those for Nano-62 and Nano-94. However, the cutoff levels were comparable, at 0.39 μg/ml (Nano-7), 0.2 μg/ml (Nano-62), and 0.2 μg/ml (Nano-94).

Initially, the Nanobody binding interactions with the GI.1 P domain and GI.1 VLPs were confirmed using a direct enzyme-linked immunosorbent assay (ELISA). The three GI Nanobodies bound to the P domain in a dose-dependent manner and with a comparable cutoff dilution (Fig. 1A). Likewise, Nano-62 and Nano-94 bound to the VLPs in a similar dose-dependent manner, whereas Nano-7 had an approximate 2-fold lower optical density at 490 nm (OD_490_) signal. Nevertheless, the three Nanobodies bound at a similar cutoff dilution (Fig. 1B). These results showed that all Nanobodies interacted with the P domain and were capable of detecting intact VLPs. To determine if the Nanobodies were cross-reactive against other GI genotypes, a direct ELISA was performed with GI.2, GI.3, GI.4, and GI.11 VLPs. All three Nanobodies were GI.1 specific and did not cross-react with other GI genotypes.

### Thermodynamic properties of GI Nanobodies.

**FIG 2:**
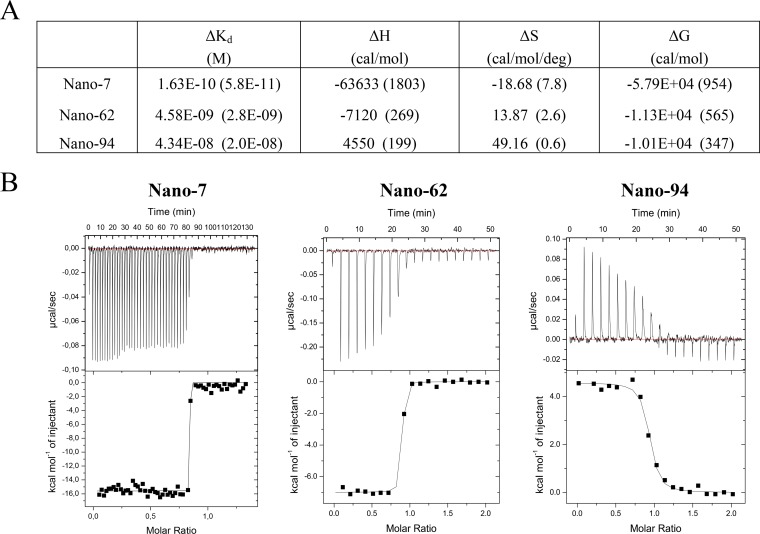
Nanobody binding affinities. (A) Thermodynamic constants (dH, enthalpy change; dS, entropy change; dG, Gibbs free energy change; *K_d_*, binding affinity) are summarized in the table. (B) ITC was performed to evaluate the thermodynamic parameters of the interaction between GI.1 P domain and Nanobodies. Examples of the titrations (upper graphs) are shown. The binding isotherm was calculated using a single binding site model after subtraction of the heat of dilution (lower graphs). All three Nanobodies exhibited nanomolar binding affinities (subnanomolar for Nano-7) and 1:1 stoichiometry. Nano-7 and Nano-62 showed exothermic binding, whereas Nano-94 bound endothermically. All binding reactions were characterized with an exothermic type of reaction.

The thermodynamic properties of Nanobody binding to the GI.1 P domain were analyzed using isothermal titration calorimetry (ITC) (Fig. 2). Two Nanobodies exhibited binding with nanomolar affinities, where Nano-62 had a *K_d_* (dissociation constant) of 4.58 × 10^−9^ M and Nano-94 − *K_d_* was 4.34 × 10^−8^ M. Nano-7 showed a subnanomolar *K_d_* of 1.63 × 10^−10^ M. The binding reaction of Nano-7 to the P domain was exothermic and driven by a very large enthalpy change, while unfavorable entropy input was less significant. The exothermic binding of Nano-62 was characterized by a large enthalpy change coupled with favorable entropy of the binding reaction (Fig. 2). On the contrary, Nano-94 binding was endothermic and was associated with a positive enthalpy change and large positive entropy. Analysis of stoichiometry indicated binding of one Nanobody molecule per GI P domain monomer (*n* = ∼1). Overall, the thermodynamic properties of these GI Nanobodies resembled those of previously characterized GII Nanobodies.

### X-ray crystal structures of GI.1 P domain-Nanobody complexes.

In order to determine the precise binding sites, the structure of Nanobodies in complex with the GI.1 P domain was determined using X-ray crystallography. Data statics of the complexes are shown in Table 1. The GI.1 P domain comprised residues 226 to 278 (P1-1) and 406 to 520 (P1-2), whereas the P2 subdomain was located between residues 279 and 405. The overall structure of the P domains in all Nanobody complex structures was reminiscent of the unbound P domain (12). The electron densities for all Nanobodies were well resolved and had the typical immunoglobulin fold. The binding sites of Nano-7 and Nano-62 were similarly located on the side of the P domain, whereas Nano-94 bound on the top.

**TABLE 1:** Data collection and refinement statistics of human norovirus GI.1 P domain complex structures^*a*^

Parameter	GI.1 and Nano-7 (PDB ID 6H6Y)	GI.1 and Nano-62 (PDB ID 6H6Z)	GI.1 and Nano-94 (PDB ID 6H71)
Data collection			
Space group	P2_1_	C2	C2
Cell dimensions			
*a*, *b*, *c* (Å)	59.17, 140.90, 92.15	172.24, 89.64, 61.92	94.89, 111.06, 122.46
α, β, γ (**°**)	90, 91.81, 90	90, 107.80, 90	90, 99.81, 90
Resolution range (Å)	47.53–1.58 (1.63–1.58)	46.67–2.09 (2.16–2.09)	49.40–2.31 (2.40–2.31)
*R*_merge_ (%)	5.53 (47.41)	7.1 (55.40)	5.04 (54.33)
*I*/σ*I*	11.70 (2.05)	11.20 (1.70)	14.21 (2.08)
Completeness (%)	97.59 (94.62)	96.90 (82.80)	97.19 (92.74)
Redundancy	3.4 (3.3)	4.0 (3.9)	2.9 (2.9)
Refinement statistics			
Resolution range (Å)	47.53–1.58	46.67–2.09	49.40–2.31
No. of reflections	201,442	51,566	53,108
*R*_work_/*R*_free_ (%)	15.46/17.48	17.83/21.74	19.25/21.71
No. of atoms	12,985	6,328	6,125
Protein	11,987	6,079	5,980
Ligand/ion	26	30	16
Water	972	219	129
Avg *B* factors (Å^2^)			
Protein	23.49	48.38	57.76
Ligand/ion	34.55	46.80	54.12
Water	29.99	44.15	50.23
Ramachandran plot (%)			
Most favorable	97.42	97.54	98.12
Allowed	2.58	2.46	1.88
Outliers	0	0	0
RMSD			
Bond lengths (Å)	0.005	0.008	0.002
Bond angles (**°**)	1.190	0.900	0.620

a Each data set was collected from a single crystal. Values in parentheses are for the highest-resolution shells. RMSD, root mean square deviation.

### Crystal structure of the GI.1 P domain–Nano-7 complex.

**FIG 3:**
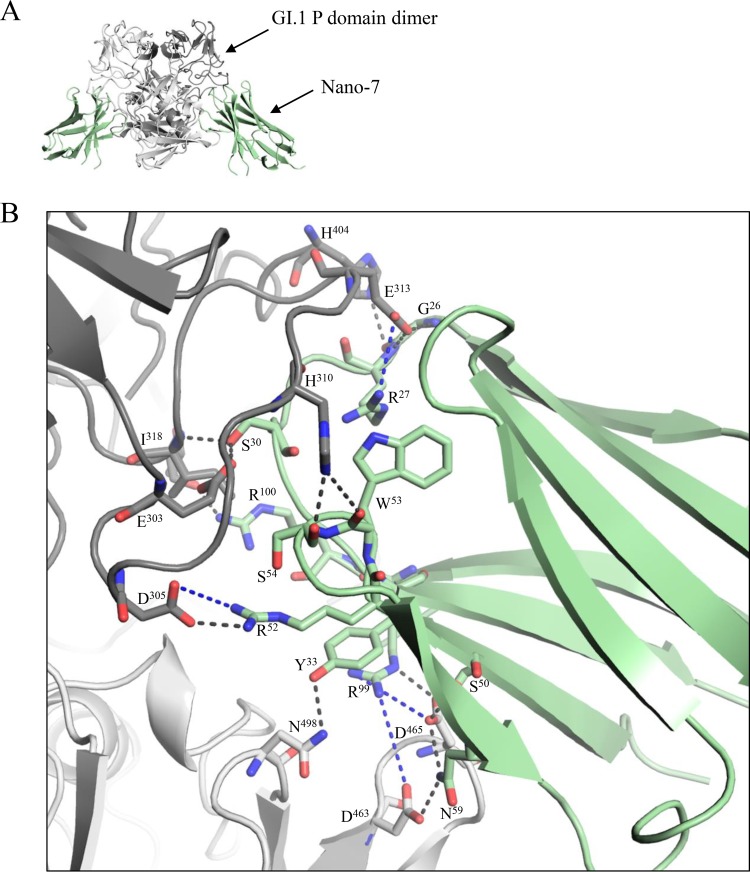
X-ray crystal structure of GI.1 P domain Nano-7 complex. (A) The asymmetric unit contained one P domain dimer (gray) and two Nano-7 molecules (green). Nano-7 bound to the side of the P domain. (B) Close-up of the P dimer and Nano-7 interacting residues. The P domain hydrogen bond interactions included side-chain and main-chain interactions from both monomers. Direct hydrogen bonds were formed with P domain chain A-Nano-7, H310-S54, E313-R27, D305-R52, I318-S30, E303-S30, T280-R100, Q449-D105, H404-G26, Q449-T103, E313-G26, and H310-W53; and chain B, D465-S50, D463-N59, N498-Y33, D465-R99, and D465-N59. Electrostatic interactions formed between P domain chain A-Nano-7, F312-R27, D305-R52, R275-D105, E313-R27, R275-D105; and chain B, D463-R99 and D465-R99. Hydrophobic interactions involved P domain chain A-Nano-7, V282-L101, L276-R100, A446-L101, L276-L101, and F312-R27; and chain B, P237-A102, P464-A102, V500-A102, and P237-L101.

**FIG 4:**
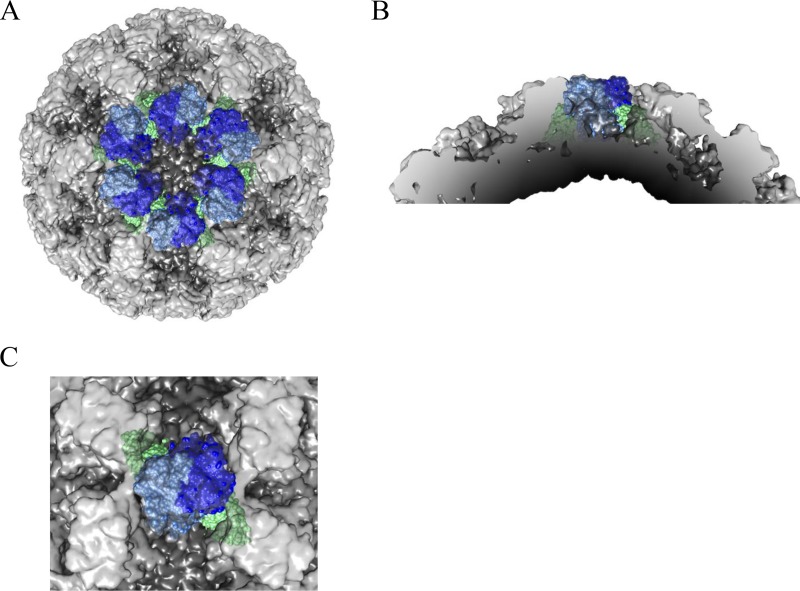
Model of Nano-7 binding on intact particles. (A) The X-ray structure of the GI.1 P domain and Nano-7 complexes fitted into the X-ray structure of the GI.1 VLP (PDB ID 1IHM). The VLPs are shaded light gray (P domain) and dark gray (S domain), while the GI.1 P domain and Nano-7 are colored blue and green, respectively. (B) Close-up of the 3-fold icosahedral axis shows Nano-7 clashing with neighboring P domains. (C) Cross-section showing that Nano-7 clashes with neighboring P domains and the adjoining S domain.

The crystal structure of GI.1 P domain–Nano-7 complex was solved to 1.58-Å resolution. Nano-7 bound on the side of the P domain in a grove between two P domain monomers (Fig. 3A). Nano-7 interacted with P1 and P2 subdomain residues as well as both P domain monomers, which included both main and side chains (Fig. 3B). Seven P2 subdomain and four P1 subdomain residues interacted with Nano-7, forming a vast network of direct hydrogen bonds. In addition to hydrogen bonds, electrostatic and hydrophobic interactions with the P1 and P2 subdomain residues were also observed. Overall, Nano-7 was held with copious P domain residues (21 residues and 33 interactions), the most observed to date for any norovirus-specific Nanobody (12, 13). Indeed, this finding was likely related to the subnanomolar affinity of Nano-7 (Fig. 2). Interestingly, Nano-7 bound in such a way that the end opposite to the complementarity-determining region (CDR) loops of the Nanobody might contact the S domain and neighboring P domains. Superposition of GI.1 P domain–Nano-7 complex onto the GI.1 VLP structure showed that the Nano-7 clashed with the S domain as well as neighboring P domains (Fig. 4). This result suggested that the neighboring P domains and/or the S domain would need to move in order for Nano-7 to bind to intact particles, as observed in the ELISA (Fig. 1).

### Crystal structure of the GI.1 P domain–Nano-62 complex.

**FIG 5:**
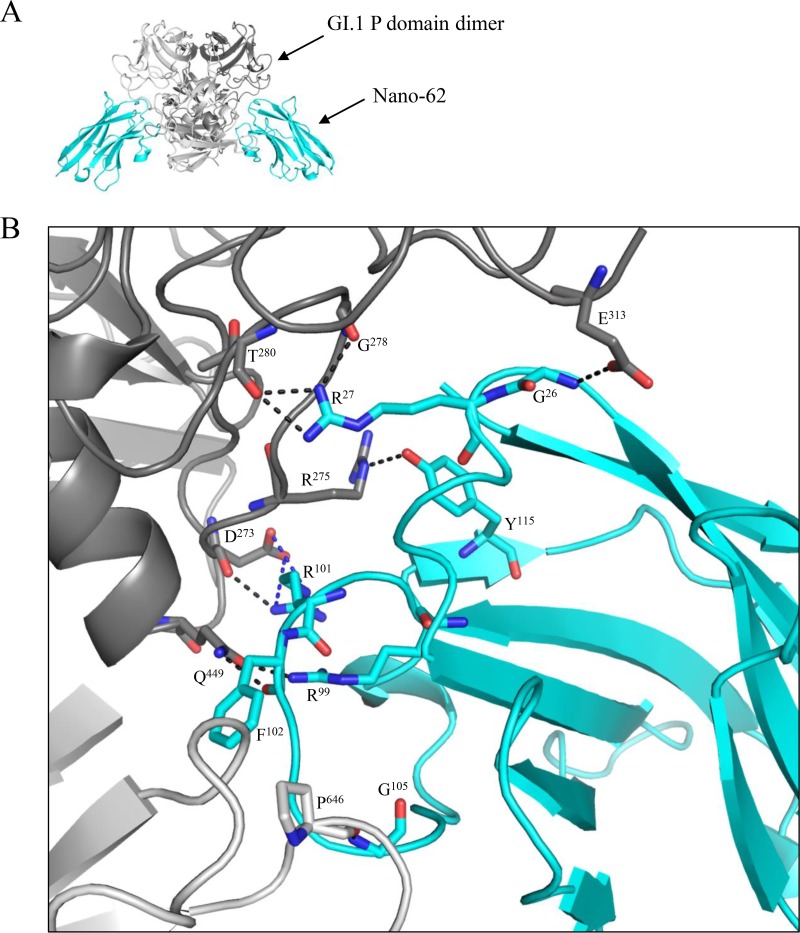
X-ray crystal structure of GI.1 P domain Nano-62 complex. (A) The asymmetric unit contained one P domain dimer and two Nano-62 molecules (cyan). Nano-62 bound to the side of the P domain. (B) Close-up of the P dimer and Nano-62 interacting residues. The P domain hydrogen bond interactions included side-chain and main-chain interactions from both monomers. Direct hydrogen bonds were formed with P domain chain A-Nano-62, P464-G105 and D465-R52; and chain B, Q449-R99, E313-G26, G278-R27, T280-R27, R275-Y115, D273-R101, and Q449-F102. Electrostatic interactions formed between P domain chain A-Nano-62, D465-R52; and chain B, D273-R101. Hydrophobic interactions involved P domain chain A-Nano-62, P237-F102 V500-F102, V462-V104, P464-F102, and P464-V104; and chain B, H404-S25 and R275-R101.

**FIG 6:**
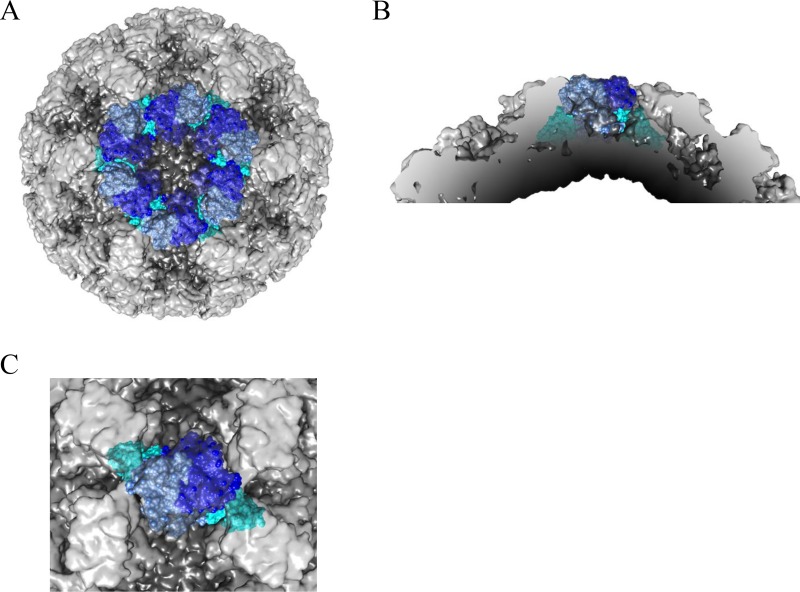
Model of Nano-62 binding on intact particles. (A) The X-ray structure of the GI.1 P domain and Nano-62 complexes fitted into the X-ray structure of the GI.1 VLP (PDB ID 1IHM). The VLPs is shaded light gray (P domain) and dark gray (S domain), while the GI.1 P domain and Nano-62 are colored blue and cyan, respectively. (B) Close-up of the 3-fold icosahedral axis shows Nano-62 clashing with neighboring P domains. (C) Cross-section showing that Nano-62 clashes with neighboring P domains and the adjoining S domain.

The structure of GI.1 P domain–Nano-62 complex was solved to 2.09-Å resolution. Similar to Nano-7, we found that Nano-62 bound on the side of the P domain and in the grove between two P domain monomers (Fig. 5A). However, compared to Nano-7, fewer direct hydrogen bonds were observed, and most (7 of 10) were contributed with P1 subdomain residues (Fig. 5B). Likewise, most (8 of 9) of the electrostatic and hydrophobic interactions were provided with P1 subdomain residues (Fig. 5B). Overall, the P domain interacted with Nano-62 with 10 residues from P1 subdomain and only 3 residues from P2. In contrast, for Nano-7, about half of the total (9 out of 21) interacting residues were located in P2. Interestingly, Nano-62 was also orientated in a way that one site of the Nanobody clashed with the S domain and neighboring P domains (Fig. 6). Surprisingly, this finding was similar to Nano-7 (Fig. 4). Moreover, several GII Nanobodies were shown to bind in occluded regions on the capsid (13, 20). However, in the case of the GII VLPs, the P domain was raised off the S domain, which might allow Nanobodies to squeeze into the region between the P and S domains.

### Crystal structure of the GI.1 P domain–Nano-94 complex.

**FIG 7:**
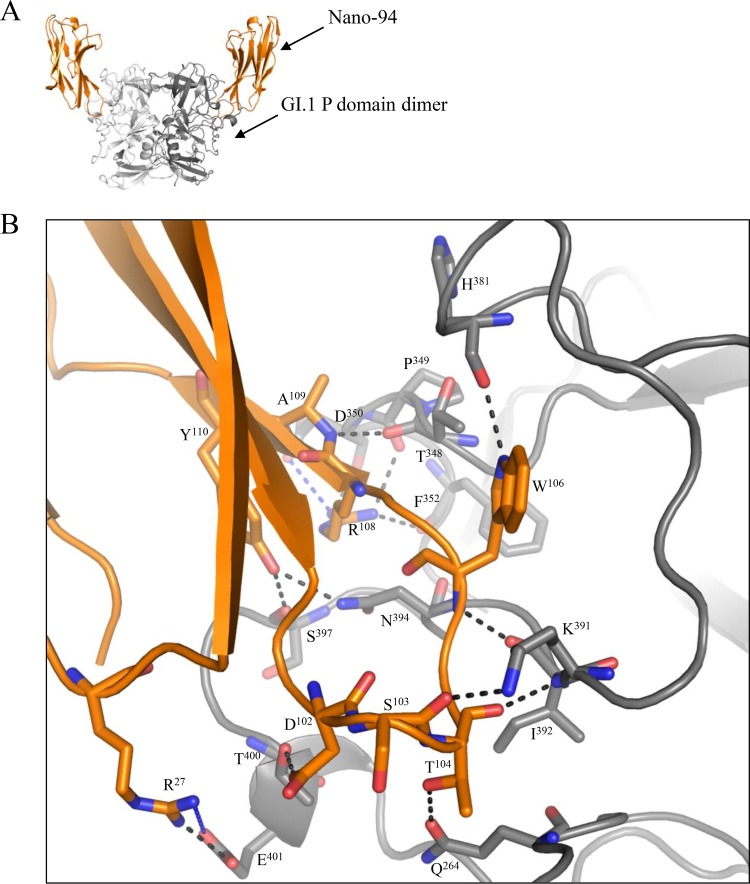
X-ray crystal structure of GI.1 P domain Nano-94 complex. (A) The asymmetric unit contained one P domain dimer and two Nano-94 molecules (orange). Nano-94 bound to the top of the P domain. (B) Close-up of the P dimer and Nano-94 interacting residues. The P domain hydrogen bond interactions included side-chain and main-chain interactions from both monomers. Direct hydrogen bonds were formed with P domain chain A-Nano-94, Q264-T104, K391-S103, S397-Y110, I392-W106, T348-A109, T400-D102, F352-R108, I392-T104, E401-R27, N394-Y110, H381-W106, P349-R108, and D350-R108. Electrostatic interactions formed between P domain chain A-Nano-94, D350-R108 and E401-R27. Hydrophobic interactions involved P domain chain A-Nano-94, P382-F107, H381-Y98, P379-W106, and I392-I105.

The structure of GI.1 P domain–Nano-94 complex was solved to 2.31-Å resolution. Nano-94 bound on the top of the P domain and interacted with one P domain monomer (Fig. 7A). Direct hydrogen bonds as well as electrostatic and hydrophobic interactions were observed (Fig. 7B). All binding residues, except one (Asp102), were located on the P2 subdomain. Interestingly, the Nano-94 binding pocket was in close proximity to the GI.1 HBGA pocket but lacked any common binding residues (33).

### Alignment of capsid residues interacting with Nanobodies.

**FIG 8:**
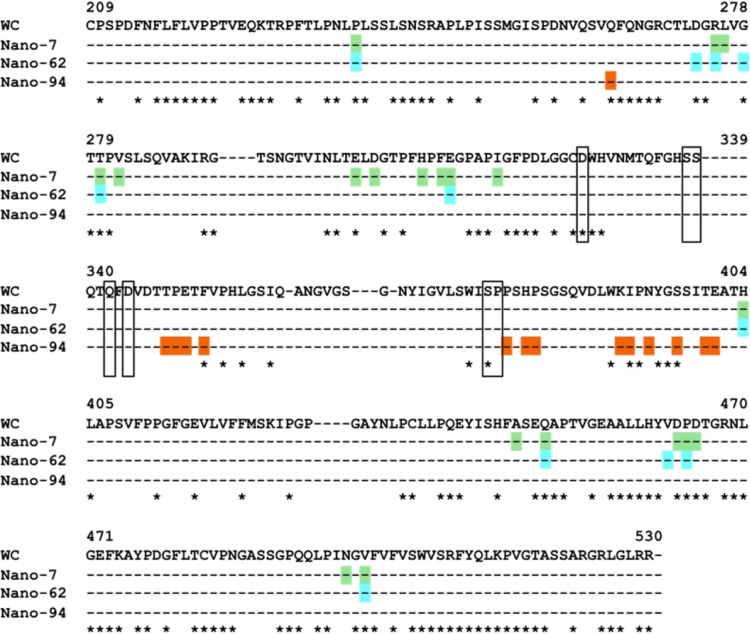
Capsid sequence alignment of GI genotypes. Seven different GI genotype capsid sequences were aligned using ClustalX. The GI.1 West Chester (GenBank accession no. AY502016.1) capsid sequence was used as the consensus sequence; other sequences include GI.1 Norwalk virus (GenBank accession no. M87661.1), GI.2 258 (GenBank accession no. AB078335), GI.3 645 strain (GenBank accession no. BD011871), GII.4 CV strain (GenBank accession no. AB042808), GI.8 WUG1 strain (GenBank accession no. AB081723), and GI.11 #8 strain (GenBank accession no. AB058547). For clarity, only GI.1 West Chester residues are shown. The GI.1 residues interacting with Nano-7 (green), Nano-64 (cyan), and Nano-94 (orange) are colored accordingly. The asterisks mark conserved amino acids. The GI.1 P domain residues interacting with HBGAs are boxed.

In order to better understand why the GI.1 Nanobodies were genotype specific, we aligned capsid sequences of other GI genotypes (Fig. 8). Overall, the GI capsid sequences were rather variable compared to GII sequences (3, 34). The Nanobody binding residues were all located in regions of the P domain, which included a mixture of both conserved and variable residues. This finding corresponded well with the lack of Nanobody cross-reactivity among other GI genotypes. In addition, this discovery suggested that the lack of capsid conservation likely limited the ability to identify cross-reactive GI Nanobodies, as was observed in other MAb studies (11, 35).

### HBGA-blocking assay.

**FIG 9:**
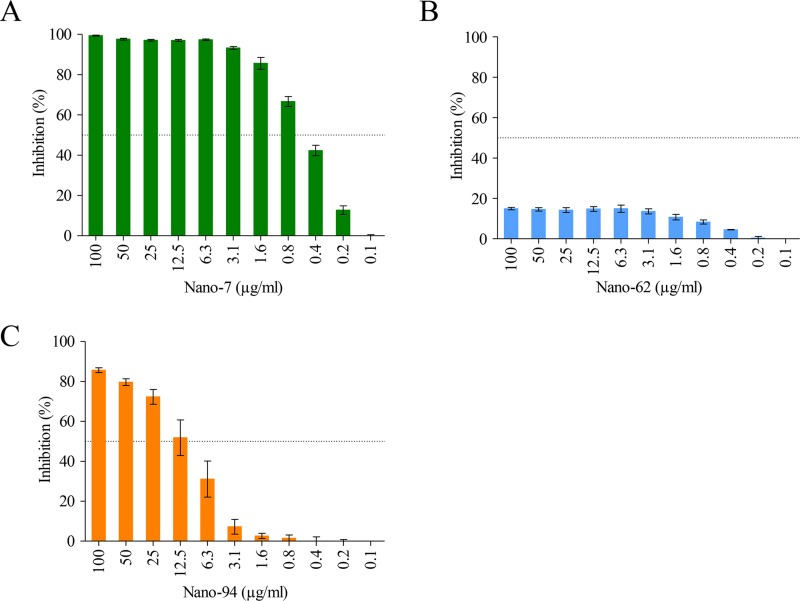
Nanobody inhibition of VLP binding to PGM. In the HBGA-blocking assay, serially diluted Nano-7 (A), Nano-62 (B), or Nano-94 (C) was preincubated with GI.1 VLPs and then added to the PGM-coated wells. Nano-7 and Nano-94 blocked the binding of GI.1 VLPs to PGM in a dose-dependent manner, with IC_50_s of 0.43 μg/ml and 9.23 μg/ml, respectively. Nano-62 showed weak inhibition, with a maximum inhibition of 15%. The experiments were performed in triplicate (error bars shown).

Our previous study showed that GII Nanobodies could block attachment to porcine gastric mucin (PGM) (20). Consequently, we evaluated whether these GI.1 Nanobodies also blocked VLP attachment to PGM using a similar blocking assay (Fig. 9). We found that Nano-62 weakly blocked VLP attachment to PGM, whereas both Nano-7 and Nano-94 blocked GI.1 VLPs in a dose-dependent manner, with half-maximal inhibitory concentrations (IC_50_s) of 0.4 μg/ml and 9.2 μg/ml, respectively. These results were similar to those with GII Nanobodies, where not all Nanobodies were capable of blocking, despite binding at similar regions on the P domain (20). It is tempting to speculate that the greater number P2 subdomain residues interacting with Nano-7, compared to Nano-62, influenced the ability of Nano-7 to inhibit binding to HBGAs, although direct evidence is lacking. Another mechanism might relate to allosteric inhibition, since a similar finding was observed with several GII-specific Nanobodies (20).

### VLP structural integrity upon Nanobody treatment.

**FIG 10:**
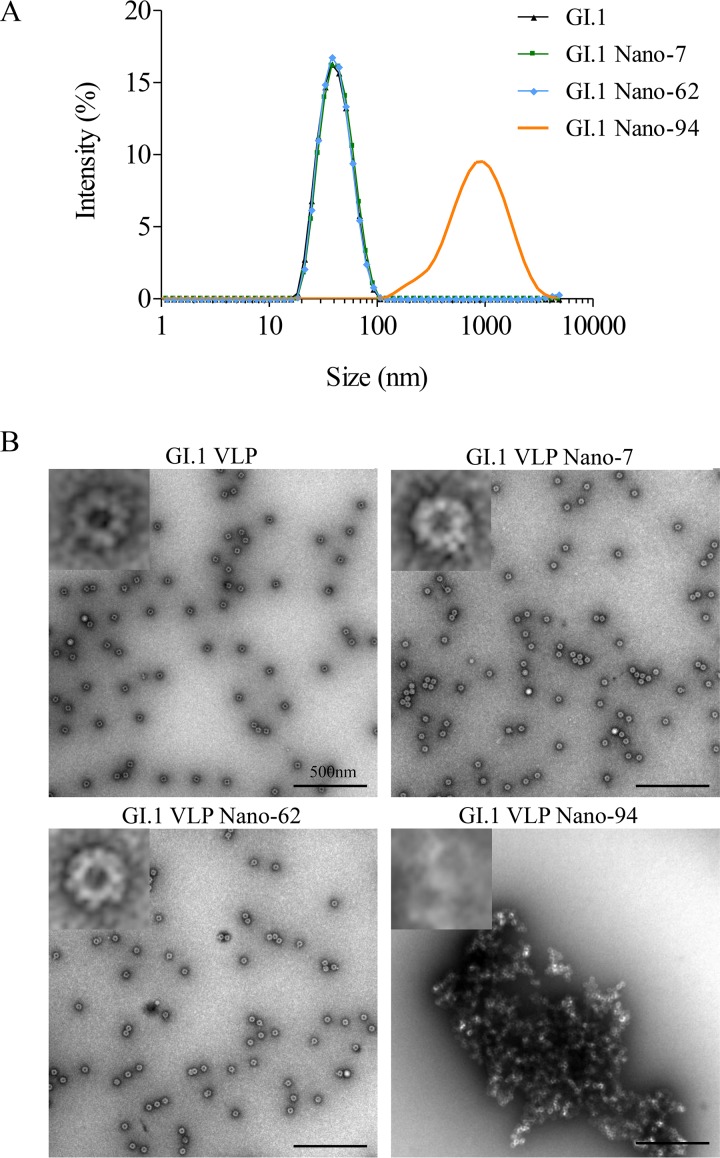
VLPs after Nanobody treatment. **(**A) DLS was used to evaluate the hydrodynamic size of GI.1 VLPs after Nanobody treatment. Incubation of GI.1 VLPs with Nano-94 (orange) led to a considerable diameter increase and particle aggregation compared to those with GI.1 VLPs alone (black). Nano-7 (green) and Nano-62 (cyan) treatment did not affect the particle sizes. Experiments were performed with native-size (T=3) GI.1 Norwalk virus VLPs. (B) EM analysis of untreated and Nanobody-treated VLPs. Nano-94 treatment produced large aggregates of intact native-size VLPs, whereas Nano-7 and Nano-62 had no obvious changes.

In order to evaluate if the GI Nanobodies might alter the stability of intact VLPs, we measured the diameters of Nanobody-treated VLPs using dynamic light scattering (DLS). The untreated VLPs exhibited a single peak, indicating a homogenous sample with VLP diameter of 42 nm (Fig. 10A). Incubation of VLPs with Nano-94 dramatically increased the heterogeneity and led to a peak shift to ∼1,000 nm, suggesting that Nano-94 treatment caused particle aggregation. In contrast, Nano-7 or Nano-62 treatment did not affect the VLP diameter (Fig. 10A).

To directly observe the effects, the Nanobody-treated VLPs were examined using EM (Fig. 10B). After treatment with either Nano-7 or Nano-62, the VLP morphology remained comparable to that of untreated VLPs, whereas for Nano-94 treatment, we observed large aggregates of VLPs. These results confirmed the DLS data. Moreover, the electron microscopy (EM) results suggested that particle aggregation likely blocked VLP binding to PGM.

### Combination of GI Nanobody and 2′FL treatment.

**FIG 11:**
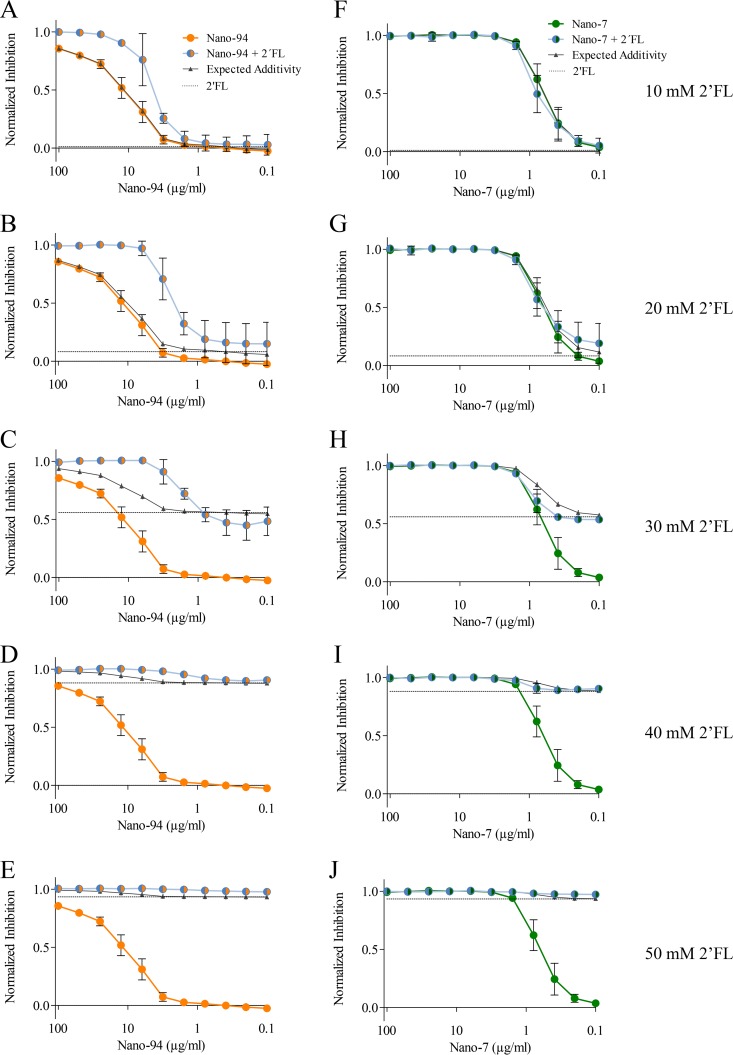
Treatment of Nano-7 or Nano-94 with 2′FL. (A–E) Serially diluted Nano-94 was combined with constant concentrations of 2′FL. The graph shows Nano-94 alone (orange line), 2′FL alone (dashed black line) a combination of Nano-94 and 2′FL (blue line), and expected values for additivity according to the Bliss model (black line). Nano-94–2′FL treatment shows synergistic effects. (F to J) Serially diluted Nano-7 was combined with constant concentrations of 2′FL. Each graph shows normalized inhibition of GI.1 binding to PGM through Nano-7 alone (green line), 2′FL alone (dashed black line), or a combination of Nano-7 and 2′FL (blue line).

Our earlier study showed that 2′FL inhibited GI.1 VLPs from binding to PGM, with an IC_50_ of 50 mM (12). Therefore, we examined the possibility that a combination of Nanobody and 2′FL might have an enhanced HBGA-blocking potential. For these PGM-blocking assays, the VLPs were preincubated with serially diluted Nano-7 or Nano-94 combined with a constant 2′FL concentration (10, 20, 30, 40, and 50 mM 2′FL) (Fig. 11). The combination of Nano-7 or Nano-94 with 2′FL led to enhanced inhibition, indicating that no adverse effects occur between Nanobodies and 2′FL. Moreover, the extent of inhibition enhancement correlated with increasing 2′FL concentrations, suggesting a synergistic effect (Fig. 11A to C). Therefore, the mathematical model of Bliss independence (36) was used to determine if the Nanobodies and 2′FL acted in an additive or synergistic manner. The combination of Nano-94 and 40 mM or 50 mM 2′FL was not defined as synergistic, since 2′FL alone already showed high inhibition, and the calculated values for expected additive effects exceeded full inhibition (Fig. 11D and E). According to our 20% criterion for synergistic effects, our data indicated synergism at various Nano-94 and 2′FL concentrations, for example, at 10 mM 2′FL combined with 25, 12.5, and 6.3 μg/ml of Nano-94 or at 20 and 30 mM 2′FL combined with 12.5 to 3.1 μg/ml of Nano-94. The strongest synergistic effect was observed for combinations with 20 mM 2′FL. The differences between expected additivity and the observed synergistic values were statistically significant (*P* ≤ 0.05), as determined by paired *t* test and two-way analysis of variance (ANOVA).

For Nano-7, the observed inhibition of the combinations ranging from 100 to 1.6 μg/ml Nano-7 combined with 10 to 50 mM 2′FL closely matched the expected value for addition of the two single effects (Fig. 11F to J). This result indicated that a combination treatment of Nano-7 and 2′FL effect was additive.

### Combination of GII Nanobody and 2′FL treatment.

**FIG 12:**
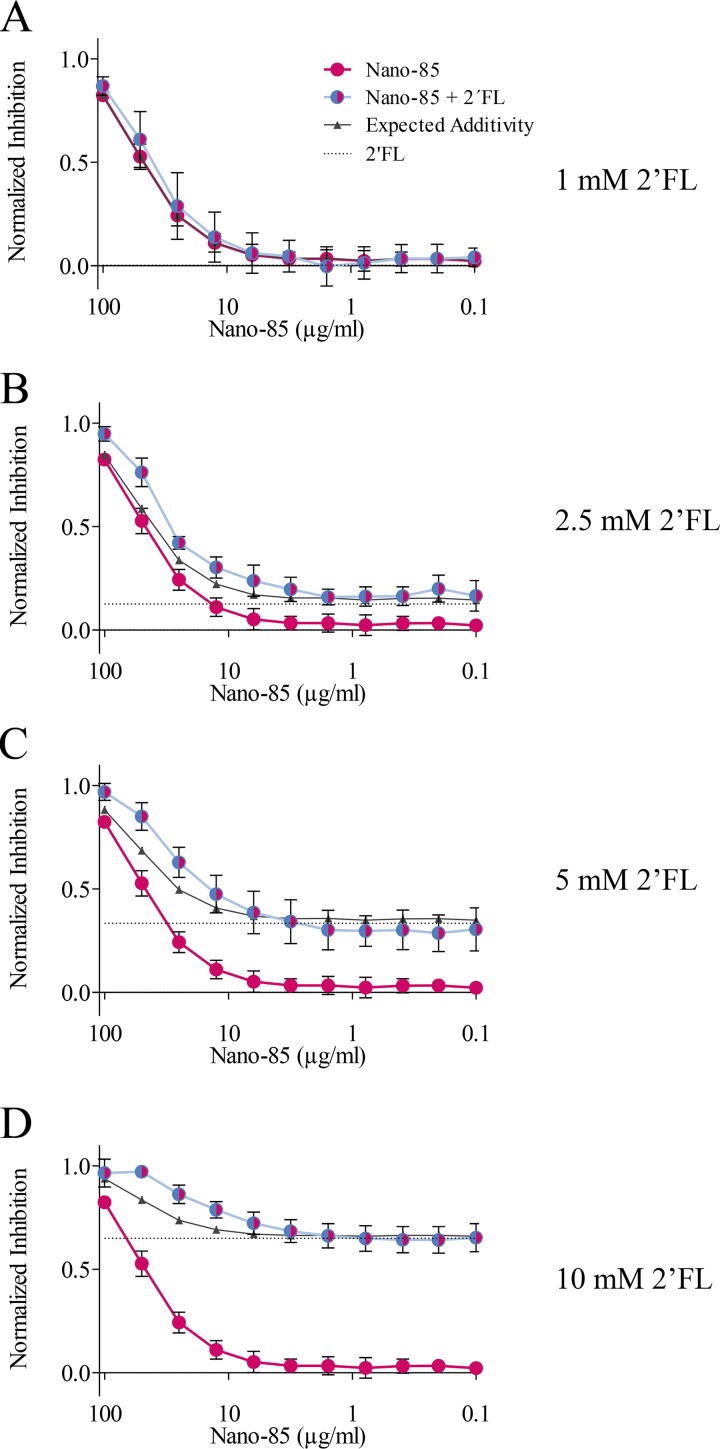
Treatment of Nano-85 with 2′FL. (A to D) Serially diluted Nano-85 was combined with constant concentrations of 2′FL. Each graph shows normalized inhibition of GI.1 binding to PGM by Nano-85 alone (pink line), 2′FL alone (dashed black line), or a combination of Nano-85 and 2′FL (blue line). The expected values for additivity according to the Bliss model (black line) were calculated for each graph. Nano-85–2′FL treatment shows additive inhibition.

To analyze whether the positive effects of a Nanobody and 2′FL combination were genogroup dependent, we repeated the combination inhibition assay with GII VLPs treated with GII-specific Nano-85 (13) and 2′FL (Fig. 12). For this assay, the 2′FL concentration was reduced to 1 to 10 mM, since the IC_50_ of 2′FL for GII.10 VLPs was 5.5 mM. Serially diluted Nano-85 was mixed with 1.0, 2.5, 5.0, and 10 mM 2′FL. The combination of Nano-85 and 1.0 mM 2′FL did not increase the inhibition effect compared to that with Nano-85 alone, suggesting that this concentration of 2′FL was too low. The combination of Nano-85 and 2.5, 5, and 10 mM 2′FL inhibited the attachment of VLPs to PGM to a greater extent than with Nano-85 or 2′FL alone, indicating a positive cooperative effect. For most combinations, the observed effects matched the calculated expected additivity. According to the Bliss independence criterion, the effects were determined to be additive. Overall, these results showed that the positive effect of a combination of Nanobody and 2′FL was not genogroup specific.

## DISCUSSION

In this study, we analyzed three new GI.1-specific Nanobodies that bound on the side and top of the P domain. We showed that Nano-7 and Nano-62 bound in such a way that the opposite end of the CDRs clashed with the S domain and neighboring P domains when modeled on the VLP structure (Fig. 4 and 6). Interestingly, this phenomenon was also observed with GII Nanobodies (13, 20) and a GII diagnostic IgG MAb (35). We speculate that like GII VLPs, the GI.1 P domains have the ability to shift in order to bind these Nanobodies. Understanding how and why the P domains shift on particles could be of critical importance, since the current vaccines in clinical trials use norovirus VLPs.

Only two human norovirus VLP structures have been determined at a resolution that can clearly distinguish the P and S domains, i.e., GI.1 (an X-ray structure) and GII.10 (a cryo-EM structure) (4, 35). The major difference between the GI and GII VLP structures was how the P domains were resting on and raised (∼15 Å) off the S domains, respectively. One possible reason might relate to the technique used for preparing the VLPs. During crystallization, the P domain could be compacted on the S domain, whereas for cryo-EM, the vitreous ice layer might allow the P domains to be less compressed. Clearly, a high-resolution cryo-EM structure of GI.1 VLPs would be required to confirm if the sample preparation influenced the VLP structure. Another explanation might simply signify the unique structural difference between the two genogroups. Still, these new Nanobody binding interactions to occluded binding sites suggested that the P domains on the GI.1 VLPs were probably more flexible than previously realized.

The notion that GI and GII particles conceal vulnerable epitopes at occluded regions raises several new lines of questions. For example, is the P domain movement an advantage or disadvantage for the virus? And, what is the function of the P domain movement? We have shown that GII VLPs treated with citrate increased in particle diameter, which might make the virus noninfectious (37, 38). We have also shown that GII Nanobodies could transform native-size VLPs to smaller VLPs ∼22 nm in diameter (20). Currently, the development of long-term immunity to current VLP vaccines is unclear, and how this might relate to particle flexibility and VLP preparation requires further investigation. Perhaps locking the P domains in a defined position could confer better host immune vaccine responses?

In our previous study, we identified several GII.10-specific Nanobodies that bound to occluded epitopes, but these were still able to efficiently inhibit VLP attachment to PGM. Similarly, the GI Nanobodies Nano-7 and Nano-62 also bound to an occluded region (Fig. 3 to 6). Nano-7 also showed efficient inhibition of VLP attachment to PGM with a mechanism that apparently involved neither direct steric interference nor particle aggregation or disruption. One possible mode of inhibition could be that binding of Nano-7 to the lower region of the P domain induces a conformational change of the capsid, subsequently interfering with the ability of the VLP to bind to HBGAs.

A comparison of the binding of Nano-62 to GI.1 and Nano-26 to GII.10 showed that the binding sites were similar, yet the inhibitory properties were rather different. Nano-62 did not influence the particle structure or inhibit HBGA binding; in contrast, Nano-26 was shown to induce particle disassembly upon binding to GII.10 VLPs, therefore effectively inhibiting HBGA binding (20). These results suggested that the specific interactions, as well as the Nanobody orientation with respect to the P domain, were important in terms of how Nanobodies could influence viral integrity. This was also stressed by the fact that Nano-7, which attached to a similar binding site as Nano-62, was strongly inhibiting HBGA binding, whereas Nano-62 did not inhibit.

Nano-94 likely inhibited HBGA attachment by inducing particle aggregation. In our panel of GII Nanobodies, Nano-32 was found to cause particle aggregation as well. In this case, Nano-32 induced rearrangement of P domain loops, which subsequently led to an alteration in surface hydrophobicity. For Nano-94, only minor rearrangements of the P domain loops were observed, implying that the mode of aggregation here was different. In both cases, the binding was characterized by positive enthalpy change coupled with large positive entropy change as main contributing factor to the free energy change (Δ*G*). Since only minor structural rearrangements in the P domain occurred upon Nanobody binding, the structures of the respective complexes could be fitted very well to the X-ray structure of the GI.1 VLP (4). However, fitting of Nanobody-P domain complexes in all possible positions on the VLP structure revealed sterical clashes on the capsid. Likewise, similar steric clashes were observed for Nano-62. Overall, these results suggested that the GI.1 hinge region between the P and S domains might have intrinsic flexibility to allow Nano-7 and Nano-62 to bind to intact particles.

Another important finding in the current study was that the combination treatment of Nanobody and 2′FL improved the attachment inhibition. In the case of 2′FL and Nano-94, the treatment resulted in a synergistic inhibitory effect. This inhibition might occur by different mechanisms. For example, Nano-94 treatment led to fast and severe aggregation of VLPs, and the remaining nonaggregated VLPs were inhibited with 2′FL. Alternatively, the aggregated VLPs had a reduced number of 2′FL binding sites, and the concentration of 2′FL relative to the remaining available binding sites was higher. For 2′FL and Nano-85, our data suggested that Nano-85 inhibited HBGA attachment by compromising the stability of VLPs (13), while 2′FL inhibited the binding to HBGAs. Compared to 2′FL and Nano-94, the combination treatment of 2′FL and Nano-85 caused an additive increase in inhibition. In the case of 2′FL with Nano-7, an additive increase in inhibition also was observed. However, the reasons for the Nano-7 inhibition were not clear, except that Nano-7 bound many more P domain residues than other known Nanobodies (13, 20), which might have indirectly affected HBGA binding. Overall, these new data showed that the positive effect of the addition of 2′FL was not limited to a single mode of action of Nanobodies or to a single norovirus genogroup.

In a prophylactic setting, a Nanobody and 2′FL combination therapy might result in an increased inhibition of norovirus infection. Nanobodies have already been used for prophylactic treatment of other viruses (39). Treatments with Nanobodies are showing promising results for a number of viral infections, including HIV, influenza virus, human respiratory syncytial virus (RSV), and rotavirus. One study showed that the oral administration of a rotavirus-specific-Nanobody was effective in treating rotavirus-induced diarrhea in an animal model (40). It also showed that the Nanobodies did not interfere with the host humoral antibody response, whereas IgG antibody treatment developed a response (40). They and others concluded that passive Nanobody treatment was safe and had very high efficacy (41–43). One of the best-known examples of using Nanobodies against virus infections is for RSV (44). Researchers showed that intranasal administration of bivalent RSV-specific Nanobodies protected mice from infection. This type of treatment could provide immediate protection for the host and should certainly be further exploited with human noroviruses (13, 20). Further inhibition studies are planned with the human norovirus cell culture system.

## MATERIALS AND METHODS

### Production of norovirus P domain and VLPs.

The GI.1 P domain (Norwalk virus, GenBank accession no. M87661.1) was produced as previously described (12). Briefly, the P domain was cloned into a modified expression vector (pMal-c2X) and transformed into Escherichia coli BL21 cells, which were grown in LB medium for 2 h at 37°C. Expression was induced with 0.7 mM isopropyl thio-β-d-galactopyranoside (IPTG) (OD_600_, 0.6) for 18 h at 22°C. Cells were harvested by centrifugation and disrupted by sonication on ice. The His-tagged fusion-P domain protein was purified from a Ni column (Qiagen) and digested with HRV-3C protease (Novagen) overnight at 4°C. The cleaved P domain was separated on the Ni column and dialyzed in gel filtration buffer (GFB; 25 mM Tris-HCl [pH 7.6] and 300 mM NaCl) overnight at 4°C. The P domain was further purified by size-exclusion chromatography, concentrated to 4 mg/ml in GFB, and then stored at 4°C. For the production of norovirus VLPs, VP1 of GI.1 (GenBank accession no. AY502016.1, strain West Chester), GI.2 (GenBank accession no. AB078335, strain Funabashi258), GI.2 (GenBank accession no. L07418, strain Southampton), GI.3 (GenBank accession no. BD011871, strain Kashiwa645), GI.4 (GenBank accession no. AB042808, strain Chiba407), and GI.11 (GenBank accession no. AB058547, strain #8) was expressed in insect cells (45, 46). The capsid sequence of the GI.1 VLPs (West Chester) used to immunize the alpaca was closely related (11 amino acids difference) to the sequence of the determined Norwalk virus VLP structure (PDB ID 1IHM).

### Production of Nanobodies.

Nano-7, Nano-62, and Nano-94 were produced as previously described at the VIB Nanobody Service Facility with the approval of the ethics commission of Vrije Universiteit, Brussels, Belgium (13). Briefly, a single alpaca was injected subcutaneously with GI.1 VLPs. A VHH library was constructed and screened for the presence of antigen-specific Nanobodies. The Nanobodies were subcloned into a pHEN6C expression vector and expressed in E. coli WK6 cells overnight at 28°C. Expression was induced with 1 mM IPTG at an OD_600_ of 0.9. The Nanobodies were extracted from periplasm and the supernatant collected. Nanobodies were eluted from a Ni column after a series of washing steps and purified by size-exclusion chromatography. Nanobodies were concentrated to 2 to 3 mg/ml and stored in GFB.

### Direct ELISA.

Microtiter plates (MaxiSorp; Thermo Scientific) were coated with 100 μl/well (5 μg/ml) of GI.1 P domain or VLPs for 1 h at 37°C. Plates were washed three times with phosphate-buffered saline (pH 7.4) containing 0.1% Tween 20 (PBS-T) and subsequently blocked with 5% skimmed milk in PBS for 1 h at room temperature. Nano-62 or Nano-94 was serially diluted (1:1) with PBS, starting at 100 μg/ml, applied to the washed ELISA plates, and incubated for 1 h at 37°C. Nanobodies were detected with horseradish peroxidase-conjugated monoclonal antibody against polyhistidine (Sigma) at a dilution of 1:4,000 in PBS. Plates were washed and then developed with *o*-phenylenediamine and H_2_O_2_ (OPD buffer) in the dark for 30 min at room temperature. Finally, the reaction was stopped with 6% (vol/vol) HCl, and absorption at 490 nm (OD_490_) was measured. A cutoff limit was set at OD_490_ of >0.15, which was ∼3 times the value of the PBS negative control (3, 47).

### Isothermal titration calorimetry measurements.

Isothermal titration calorimetry (ITC) experiments were performed using an ITC-200 (Malvern, UK). Samples were dialyzed into PBS and filtered prior to the titration experiments. Titrations were performed at 35°C (Nano-7), 30°C (Nano-62), or 25°C (Nano-94) by injecting consecutive (0.5 μl for Nano-7 and 2 μl for both Nano-62 and Nano-94) aliquots of Nanobody (150 μM) into GI.1 P domain (15 μM) with 150-s intervals. The binding data were corrected for the heat of dilution and fit to a one-site binding model to calculate the equilibrium binding constant, *K_A_*, and the binding parameters, N and Δ*H* (change in enthalpy). Binding sites were assumed to be identical.

### Crystallization of norovirus P domain and Nanobody complexes.

The GI.1 P domain was mixed separately at a 1:1.4 molar ratio with the Nano-7, Nano-62, and Nano-94, and the complexes were incubated for 2 h at 25°C. Each complex was purified by size-exclusion chromatography using a Superdex-200 column and concentrated to 4.6 mg/ml. The complex crystals were grown using the hanging-drop vapor diffusion method. For GI.1 Nano-62 and GI.1 Nano-94, the mother solution contained 0.1 M sodium citrate (pH 5.5) and 20% (wt/vol) polyethylene glycol 3000 (PEG-3000) for 3 to 7 days at 18°C. For GI.1 Nano-7, crystals were grown in mother solution containing 0.17 M ammonium acetate, 0.085 M sodium citrate (pH 5.6), 15% (vol/vol) glycerol, and 25.5% (wt/vol) PEG-4000. Prior to flash-freezing in liquid nitrogen, single crystals were transferred to a cryoprotectant containing the mother liquor with or without 30% ethylene glycol.

### Data collection, structure solution, and refinement.

X-ray diffraction data were collected at the European Synchrotron Radiation Facility, France, at beamlines ID23-1, ID29, and ID30A-3 and processed with *XDS* (48). The GI.1 P domains in complex with Nano-7, Nano-62, and Nano-94 were solved using molecular replacement with the GI.1 P domain (PDB ID 2ZL5) and a previously determined Nanobody (PDB ID 4KRN) as search models in *Phaser* (49). The complex structures were refined in multiple rounds of manual model building in *Coot* (50) and refined with *Phenix* (51). Structures were validated with *Coot* and *MolProbity* (52). The PDBePISA server was used to determine binding interfaces and calculate the surface area. The binding interactions were analyzed using Accelrys Discovery Studio, with hydrogen bonding interaction distances between 2.4 and 3.5 Å and hydrophobic interaction distances between 3.9 and 5.3 Å. Figures and protein contact potentials were generated using PyMOL (https://pymol.org).

### Dynamic light scattering.

The hydrodynamic size of VLPs was analyzed using dynamic light scattering (DLS) with a Zetasizer Nano S system (Malvern). The VLPs and Nanobodies (10 μl/10 μl) were incubated for 10 min at room temperature, diluted in 1 ml of distilled water, and then measured. Measurements were performed at 25°C in three runs with 15 measurement cycles.

### Electron microcopy.

The norovirus VLP morphology (treated and untreated) was analyzed using negative-stain electron microscopy (EM), as previously described (13). Nanobodies (1 mg/ml) and VLPs (1 mg/ml) were mixed in equal volumes and incubated for 1 h at room temperature. Prior to loading on carbon-coated EM grids, samples were quickly diluted 30 times with distilled water. The grids were washed with distilled water and stained with 1% uranyl acetate. The grids were examined on a Zeiss 910 electron microscope.

### HBGA-blocking assay.

The binding of GI.1 VLPs to porcine gastric mucin (PGM) type III (containing HBGAs) was previously determined (20). For the blocking assay, microtiter plates (MaxiSorp; Thermo Scientific) were coated with 100 μl/well of PGM (10 μg/ml; Sigma) for 1 h at 37°C. The plates were washed three times with phosphate-buffered saline (pH 7.4) containing 0.1% Tween 20 (PBS-T) and subsequently blocked with 5% skimmed milk in PBS for 1.5 h at room temperature. Nano-7, Nano-62, or Nano-94 was serially diluted in PBS and then added to GI.1 VLPs (final concentration, 0.5 μg/ml) for 30 min at room temperature. The plates were washed three times with PBS-T, and then 100 μl of each VLP-Nanobody mixture was added to triplicate wells for 1 h at 37°C. After washing, 100 μl of GI.1-specific biotinylated Nanobody 60 (Nano-60) (20) was added. Following a washing step, horseradish peroxidase (HRP)-conjugated streptavidin monoclonal antibody was added to the wells and incubated for 1 h at 37°C. Plates were washed and then developed with *o*-phenylenediamine dihydrochloride (OPD) and H_2_O_2_ in the dark for 30 min at room temperature. Finally, the reaction was stopped with 3 N HCl, and the absorbance at 490 nm (OD_490_) was measured. Nano-62 (GI negative control) and 2′FL (GI positive control) were used as controls in each experiment. The OD_490_ value of the untreated VLPs was set as the reference value corresponding to 100% binding. The percentage of inhibition was calculated as [1 − (treated VLP mean OD_490_/mean reference OD_490_)] × 100. The half-maximal inhibitory concentration (IC_50_) was determined using the Prism software (version 6.0) (14). All experiments with PGM binding were performed in triplicate, and standard deviations were calculated. For the dual Nano-7/Nano-94 and 2′FL treatment, a similar approach was performed, except that the serial dilutions of Nano-7/Nano-94 were combined with constant concentrations of 2′FL (final concentrations, 10, 20, 30, 40, and 50 mM).

In a similar assay, blocking of GII.10 attachment to PGM by a combination of Nano-85 and 2′FL was examined. In this assay, incubation times and temperatures differed slightly due to the different VLPs used. Coating was performed for 4 h at room temperature, blocking was done at 4°C overnight, and the treated VLPs with Nano-85–2′FL were incubated for 2 h at room temperature. The GII.10 VLPs were detected using polyclonal anti-GII.10 rabbit antibodies (1 h at room temperature) and polyclonal anti-rabbit-HRP conjugate (1 h at room temperature) (47).

### Bliss independence calculation.

To evaluate the interaction of Nano-7 and Nano-94 with 2′FL, Bliss independence was employed for data analysis of the HBGA-blocking assays (36, 53). This model assumes a stochastic process in which both compounds contribute to the final result but act independently by targeting different sites. The expected additive effect $\left(E_{\text{Nanobody}-2\prime \text{FL}}\right)$ can be calculated based on the probability of the independent blocking activities of 2′FL and Nanobody $\left(E_{\text{Nanobody}}\;\text{and }E_{2\prime \text{FL}}\right)$ with the following equation, where 0 ≤ $E_{\text{Nanobody}}\leq 1\;\text{and}\;0\leq E_{2\prime \text{FL}}\leq 1:$

$$E_{\text{Nanobody}-2\prime \text{FL}}=E_{\text{Nanobody}}+E_{2\prime \text{FL}}–E_{\text{Nanobody}}\;\times \;E_{2\prime \text{FL}}$$

A comparison of the calculated expected additive effect to the observed results demonstrates how the drugs interact with each other. If zero interaction applies, the effects of the two drugs are additive, and as a consequence, the measured values of drug combination are close to the calculated theoretical additivity values. If the observed combination values are 20% above the expected additivity values, synergistic effects are implied, and if the observed values are 20% below the expected additivity, antagonism is implied. To determine if the difference between the expected additivity and our observed inhibition was statistically significant, we performed a paired two-tailed *t* test for all single data points and two-way ANOVA.

### Data availability.

Atomic coordinates and structure factors of the complexes were deposited in the Protein Data Bank with the accession numbers 6H6Y for the GI.1 P domain–Nano-7 complex, 6H6Z for the GI.1 P domain–Nano-62 complex, and 6H71 for the GI.1 P domain–Nano-94 complex.
